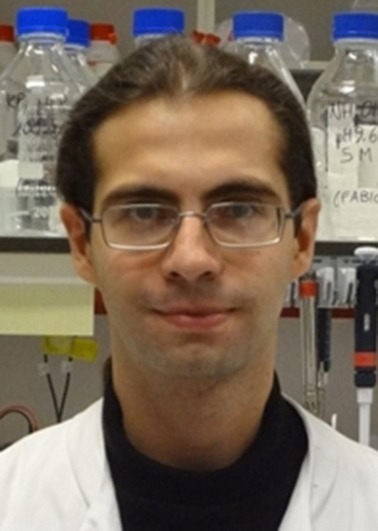# “A Study in Yellow”: Investigations in the Stereoselectivity of Ene‐Reductases

**DOI:** 10.1002/cbic.202100445

**Published:** 2021-10-13

**Authors:** Fabio Parmeggiani, Elisabetta Brenna, Danilo Colombo, Francesco G. Gatti, Francesca Tentori, Davide Tessaro

**Affiliations:** ^1^ Dipartimento di Chimica Materiali ed Ingegneria Chimica “Giulio Natta” Politecnico di Milano Piazza Leonardo da Vinci 32 20133 Milano Italy

**Keywords:** biocatalysis, enzymes, oxidoreductases, reduction, stereoselectivity

## Abstract

Ene‐reductases from the Old Yellow Enzyme (OYE) superfamily are a well‐known and efficient biocatalytic alternative for the asymmetric reduction of C=C bonds. Considering the broad variety of substituents that can be tolerated, and the excellent stereoselectivities achieved, it is apparent why these enzymes are so appealing for preparative and industrial applications. Different classes of C=C bonds activated by at least one electron‐withdrawing group have been shown to be accepted by these versatile biocatalysts in the last decades, affording a vast range of chiral intermediates employed in the synthesis of pharmaceuticals, agrochemicals, flavours, fragrances and fine chemicals. In order to access both enantiomers of reduced products, stereodivergent pairs of OYEs are desirable, but their natural occurrence is limited. The detailed knowledge of the stereochemical course of the reaction can uncover alternative strategies to orient the selectivity via mutagenesis, evolution, and substrate engineering. An overview of the ongoing studies on OYE‐mediated bioreductions will be provided, with particular focus on stereochemical investigations by deuterium labelling.

## Graphical abstract

**“Elementary, Watson!”** The recent efforts to investigate and understand the stereochemical course of the asymmetric bioreduction of C=C bonds mediated by ene‐reductases are reviewed, covering the literature of the last two decades. A general binding model is presented and successful approaches to stereodivergent reductions are discussed with representative examples.

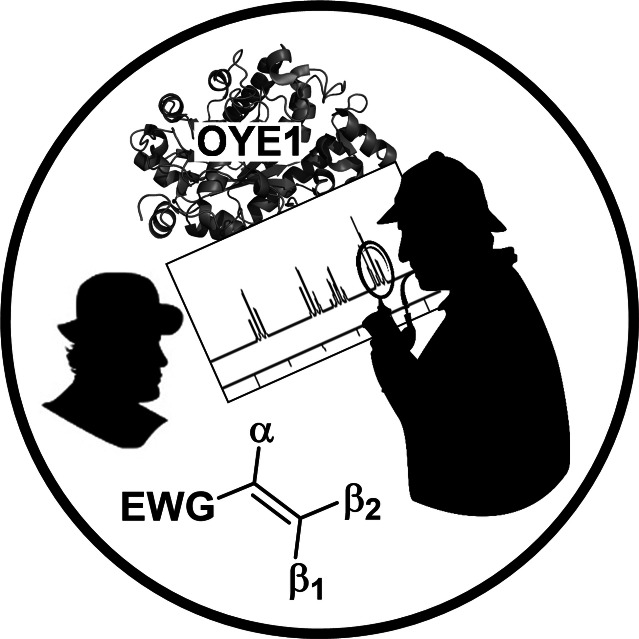

## Introduction

The asymmetric hydrogenation of C=C bonds, with the generation of up to two stereogenic centres, is a crucial transformation in the stereoselective synthesis of enantioenriched molecules, especially in the field of fine chemicals such as pharmaceuticals, agrochemicals and fragrances. Traditional chemical catalysis offers many options for this reaction, mainly based on the use of hydrogen gas and transition metal catalysts with expensive chiral ligands. A biocatalytic alternative to this transformation is the class of enzymes known as ene‐reductases (ERs), which perform C=C bond reduction, very often with extremely high enantioselectivity, at the expense of a nicotinamide cofactor (NAD(P)H) in solution without the need for dangerous hydrogen gas and toxic metals.

**Figure 1:**
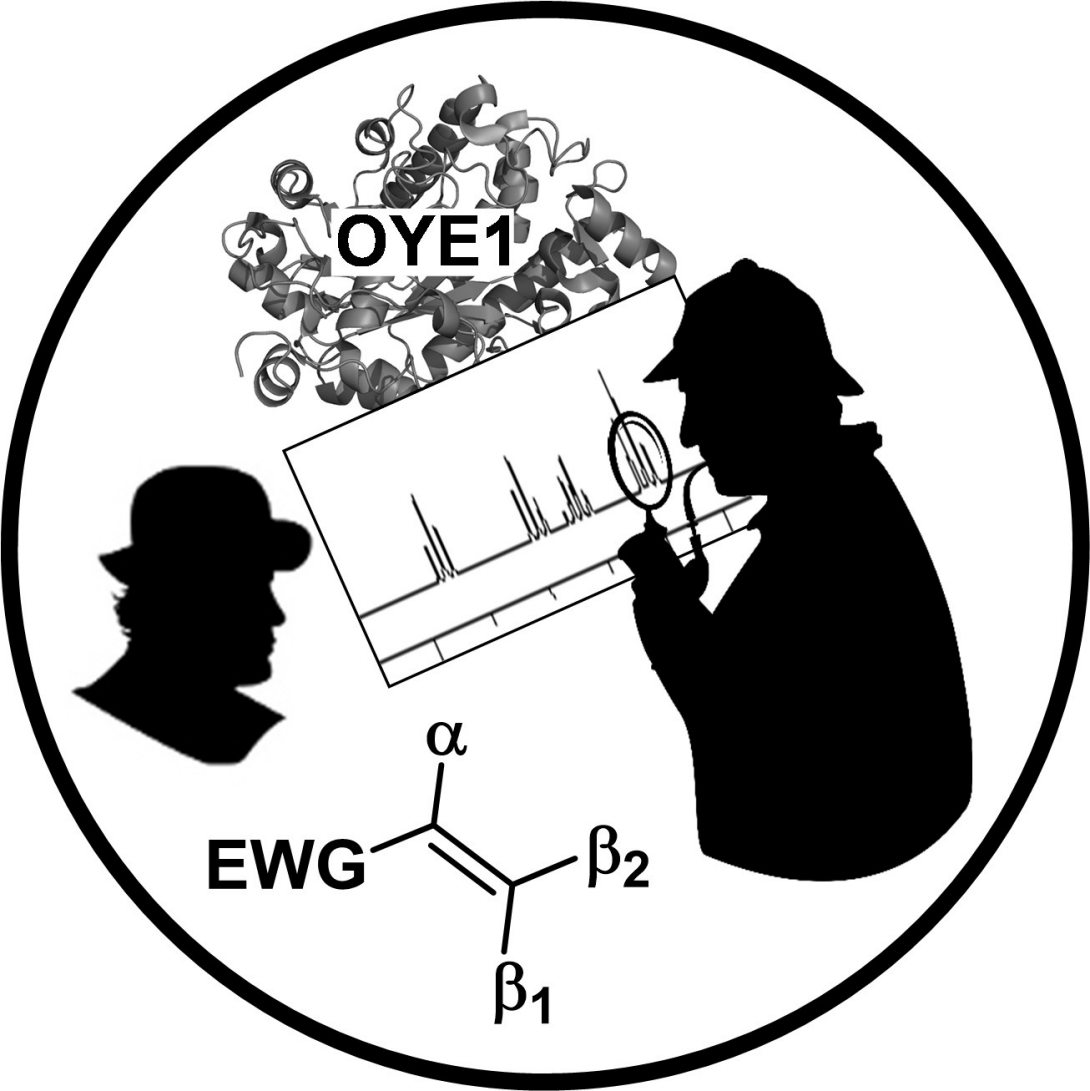
Famous detectives at work to analyse stereochemical clues.

The vast majority of ERs belongs to the well‐characterised superfamily of Old Yellow Enzymes (OYEs, E.C. 1.6.99.1), first identified in yeasts (in particular OYE1 from *Saccharomyces pastorianus*, OYE2 and OYE3 from *Saccharomyces cerevisiae*) and, shortly after, discovered also in bacteria, plants, fungi and algae. The name originates from the fact that they contain a flavin mononucleotide (FMN) prosthetic group, which is responsible for catalysis and confers an intense yellow colour to purified samples, and from their very early discovery in 1933.^[1]^ OYEs catalyse the stereoselective reduction of a broad range of C=C bonds activated by the presence of one or more electron‐withdrawing groups (EWGs, Figure 1), such as aldehydes, ketones and nitro groups, but also esters, carboxylic acids and nitriles. Notably, their physiological function is yet unclear, although they have been loosely linked to the detoxification of harmful compounds and to the response to oxidative stress.

The literature on ERs and OYEs in particular is rather vast, and a few comprehensive reviews are available,[^2^, ^3^, ^4^] to which the reader is directed for a more general perspective on the subject. Other shorter reviews covered specific topics in the field, such as novel hydride sources,^[5]^ pharmaceutical applications,^[6]^ unusual reactivities^[7]^ or phylogenetic/structural classification,^[8]^ but an updated report on stereochemical studies is missing, as of yet. Therefore, in this review, a general overview of the studies carried out in the last decade to establish the stereochemical course of C=C bioreductions will be presented, including an attempt to rationalise a large amount of literature data collected over the years in our research group as well as many others. Additionally, a brief overview of the strategies available to influence and invert the stereoselectivity of these enzymes will also be presented.

In 1887, Sir Arthur Conan Doyle published *A Study in Scarlet*, the first novel where the world‐famous investigator Sherlock Holmes appeared. The consulting detective is presented as a logical, analytical, and unbiased character, gifted with extraordinary reasoning capabilities. Furthermore, throughout the books and novels, Sherlock Holmes also displayed a profound knowledge of chemistry and biology and an unwavering belief in the collection of hard evidence, somehow anticipating modern forensic science.

Although we will never surpass or match Holmes’ outstanding abilities, the mindset and skills of this fictional character are exactly the tools required to analyse and establish with certainty the stereochemical course of ER‐mediated reductions – *“a study in yellow”* (Figure 1).

### Investigation of the Stereoselectivity of C=C Reductions Mediated by Canonical OYEs

**Scheme 1:**
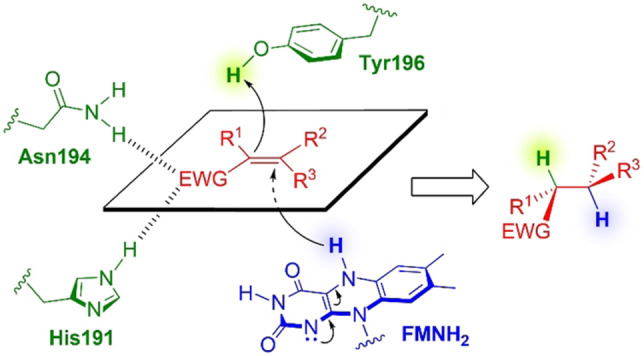
General mechanism of OYE‐mediated bioreductions of activated C=C bonds (residues numbered according to the sequence of OYE1).

The general mechanism of OYE‐mediated bioreductions has been studied extensively and has been shown to involve a two‐step reaction sequence, sketched in Scheme 1.

The EWG (one of the EWGs if the molecule bears more than one) which forms a tight H‐bond interaction with two donor residues, acts as an activating group, with the two‐fold purpose of positioning the substrate and making it more electronically reactive to the 1,4‐hydride addition. Such donor residues are His191 and Asn194 in OYE1, almost universally conserved across the family, except for a limited number of homologs which bear two His residues instead. Addition of hydride occurs from the FMNH_2_ prosthetic group to the β‐position, followed by proton transfer to the α‐position from an acidic residue nearby (Tyr196 in OYE1, also universally conserved except for at least one homolog where it is replaced by Cys). This results in formal hydrogenation of the C=C bond with *anti* stereospecificity. According to a ping‐pong bi‐bi mechanism, after the release of the product, a molecule of nicotinamide cofactor NAD(P)H subsequently binds to reduce FMN back to FMNH_2_, thus restoring the catalytically active form to restart a new reduction cycle. Therefore, in biocatalytic applications, ERs are routinely coupled with a suitable cofactor regeneration system to recycle NAD(P)^+^ using an inexpensive reducing agent (*e. g*., the combination of glucose and a glucose dehydrogenase, or formate and a formate dehydrogenase).

**Scheme 2:**
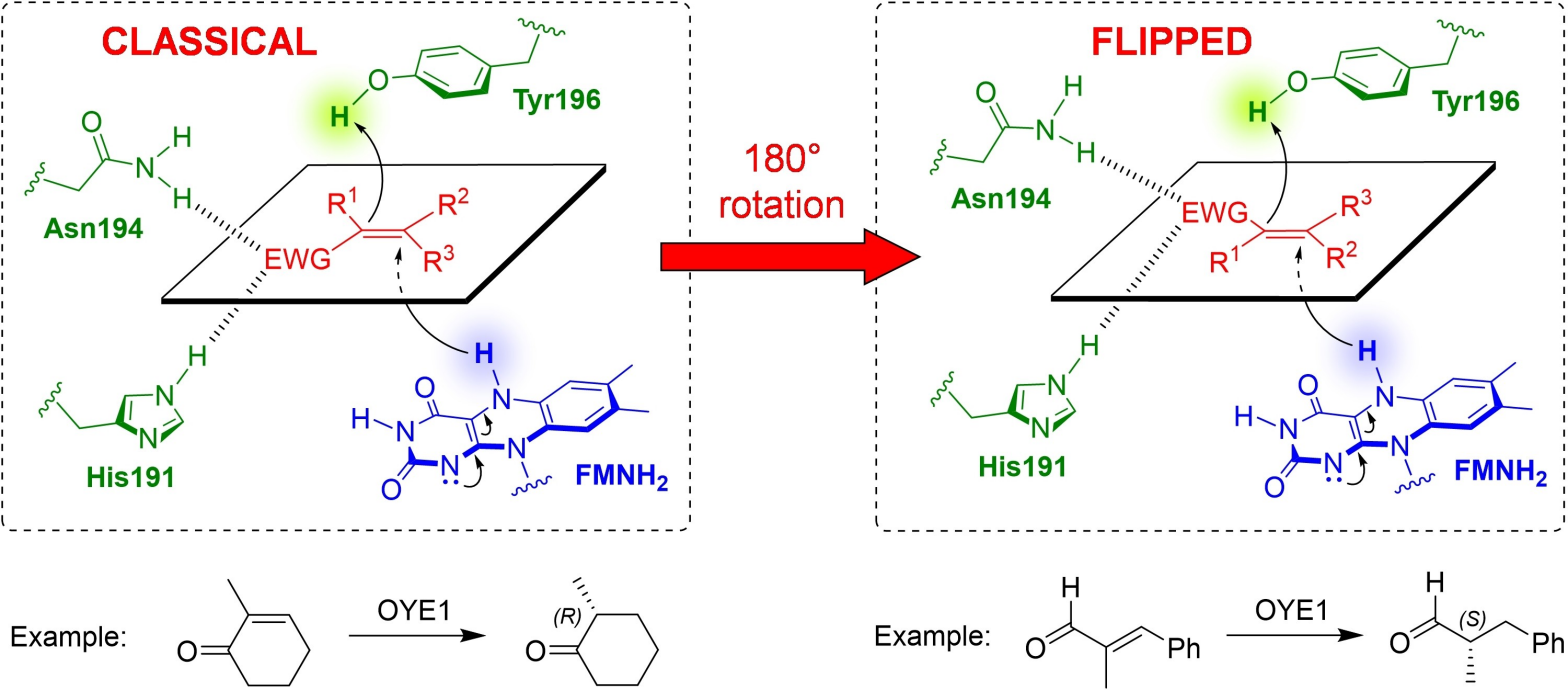
Representations of the classical and flipped binding modes (adapted from Ref. [4]).

Since the very early days, it was recognised that the stereochemical course of such reactions is not always straightforward. In particular, being the geometry of the activated C=C bond essentially planar, it has been observed that the substrate may bind to the active site of the enzyme through two different orientations that differ by an approximately 180° rotation, or “flipping”, of the substrate in the binding pocket (Scheme 2). For historical reasons, the binding mode adopted by derivatives such as α‐methylcyclohexenone has been termed “classical” binding mode, because it has been established first, while the one adopted by derivatives such as α‐methylcinnamaldehyde has been termed “flipped” binding mode.

It is apparent that, due to the structure of the active site, a prochiral substrate bound in the classical binding mode will necessarily afford products with stereochemical configuration opposite to that obtained if the same substrate is bound in the flipped mode. Examples are known of substrates or classes of substrates that either do not show a clear preference for one or the other binding mode, causing low enantioselectivity (when both binding modes may be adopted) or, as it will be discussed later, are characterised by a very fine sensitivity to the steric hindrance of the substituents (when modifying the bulkiness of the substituents causes a switch between the two modes). It also follows that, if a switch from a binding mode to the other can be “forced”, it ought to be possible to access the opposite enantiomer of the product, which is sometimes the desired and valuable one.

Many classes of substrates for different applications have been screened over the years, generating large amounts of data, and a brief overview will be provided below. Of particular interest for their broad range of synthetic applications, is the reduction of bifunctional substrates that bear more than one EWG, such as diesters, ketoesters, cyanoesters and nitroesters. Beyond their countless applications, due to the presence of multiple functional groups that can be manipulated downstream, they constitute very interesting structures to probe the enzyme‐substrate interactions, since the relative size and bulkiness of the substituents can be finely tuned by choosing appropriate groups.

**Figure 2:**
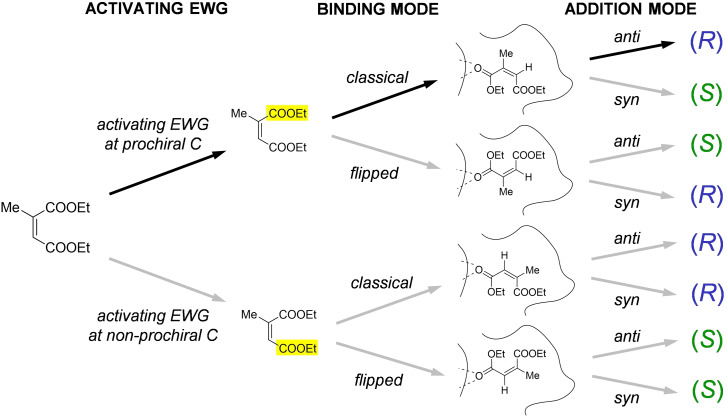
Eight possible paths for the bioreduction of diethyl citraconate. In all cases H^–^ is delivered to the β‐position from below the plane (and H^+^ to the α‐position from above the plane for *anti* addition, and from below the plane for *syn* addition). The experimentally proven option is shown as the first, indicated by black arrows.

However, in most situations, in order to understand the stereochemical course of the reaction and to identify unambiguously the binding mode, it is not sufficient to know the stereochemistry of the starting material (*E* or *Z*) and of the product (*R* or *S*). It is necessary also to establish which EWG acts as the activating one (*i. e*., binds to the active site of the enzyme) and to confirm the *anti* stereospecificity of the addition mode, ruling out *syn* addition. In particular, in the case of a generic substrate with two equal or similar EWGs, the situation is rather complex, since eight possible options have to be considered, as shown for example with the case of diethyl 2‐methylmaleate (also known as diethyl citraconate) in Figure 2.^[9]^

Crystallographic structures and/or docking studies are powerful tools to elucidate the binding, but in most cases they offer a rather “static” view of the active site, which only partially takes into account the dynamic and flexible structure of the protein, often oversimplifying the interactions on the basis of steric hindrance. This is obviously not true if very detailed computational simulations are carried out with high level of chemical accuracy, but these calculations are highly time‐ and resource‐consuming and can be performed only by experienced computational scientists.

On the other hand, the biochemist has at his disposal a surprisingly simple and powerful investigative tool that affords all the required information about the binding and mechanism of the reaction: isotopic labelling with deuterium atoms.

**Scheme 3:**
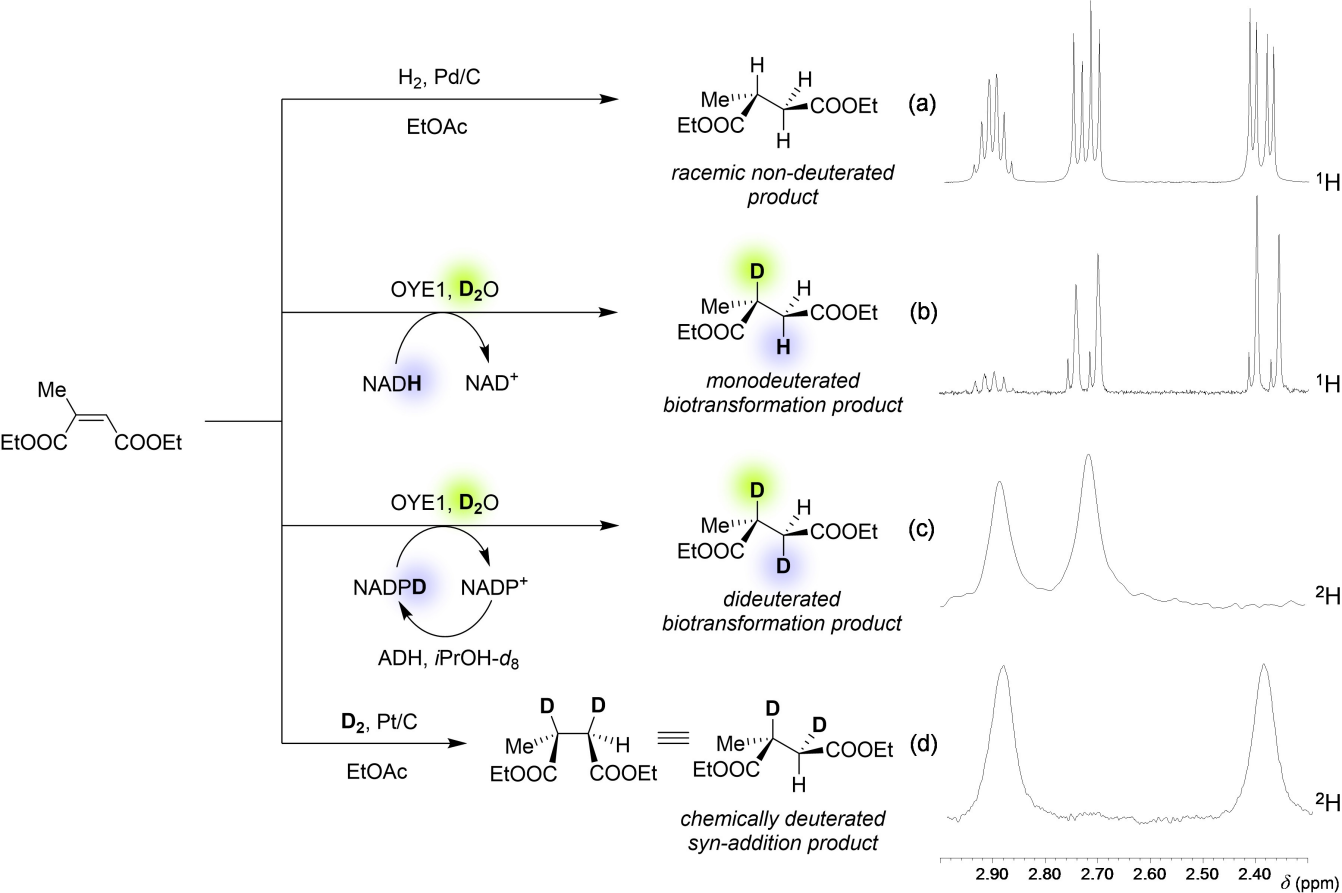
Isotopic labelling experiments to identify the activating EWG and the addition mode for diethyl citraconate as a representative example (NMR spectra adapted from Ref. [9]).

In order to establish which EWG behaves as the “activating” one (*i. e*., it binds to the H‐bond donors in the active site), it is sufficient to replace the H_2_O in the reaction medium with D_2_O. In this remarkably simple experiment, the mobile protons of acidic residues of the protein (including the catalytically relevant Tyr/Cys) are exchanged with D^+^ from the solution, and, as a result, a deuterium atom instead of a hydrogen atom is added to the α‐position with respect to the activating EWG. The comparison of the ^1^H NMR spectra of the product of such experiment with that of the reference standard of the non‐labelled racemic product allows inferring the activating EWG. In the case of diethyl citraconate, the stacked spectra of the non‐deuterated and monodeuterated products are shown in Scheme 3a, b, respectively, demonstrating clearly that the deuteration occurred at the prochiral C(2) atom of the substrate.

In order to establish the stereospecificity of the addition, a double deuteration experiment can be performed, which involves the use of D_2_O as the solvent as a source of D^+^ ions, as well as a deuterated nicotinamide cofactor NAD(P)D as a source of D^–^ for the reduction. The latter species is considerably expensive, but it can be conveniently generated *in situ* by a deuterated sacrificial substrate and a regeneration enzyme, for example isopropanol‐*d*_8_ with a suitable alcohol dehydrogenase (ADH). The analysis of ^2^H NMR spectrum of the product shows usually just two signals, proving the very high diastereoselectivity of the addition. However, this result alone is insufficient to establish which of the two possible diastereoisomers has been produced. Such spectrum needs to be compared with that of a double deuterated product obtained from a reaction with known stereochemistry, such as the reaction of the same substrate with D_2_ gas and catalytic Pt/C (which occurs stereospecifically with *syn* addition). In the case of diethyl citraconate, the ^2^H NMR spectra of the biocatalytic and chemical dideuterated products are shown in Scheme 3c, d, respectively. Their comparison demonstrates that the bioreduction occurred with *anti* addition of two deuterium atoms at C(2) and C(3). Indeed, due to the geometry of the active site, only the *anti* addition is generally possible (as illustrated in Scheme 1, which represents the typical situation), but there are examples where *syn* addition has been invoked to explain the experimental data.[^10^, ^11^] This has been ascribed to the presence of other acidic residues or even water molecules in the active site that could be able to protonate the α‐position from the opposite stereoheterotopic face.

Even in the most complex situations, having collected the results of the deuteration experiments described, it is possible to establish with certainty the stereochemical course of the reaction. In Sherlock Holmes’ words: *“When you have eliminated the impossible, whatever remains, however improbable, must be the truth.”* (A. Conan Doyle, *The Sign of the Four*). For instance, out of the eight valid hypotheses for the reduction of diethyl citraconate illustrated earlier in Figure 2, the knowledge of the configuration of the starting material (*Z*) and of the product (*R*), the identity of the activating EWG, and the addition mechanism leaves only one possibility.

Over the past decade, many such experiments have been carried out by us and several other research groups, in the attempt to explore and probe the substrate scope of these remarkably versatile biocatalysts. A representative selection of substrate classes that are well accepted (with high stereoselectivity and conversion) by OYEs is reported in Table 1.

**Table 1:** Representative examples of different groups of substrates accepted by canonical OYEs with high conversion and enantioselectivity, classified according to the model presented in Figure 3, which takes into account the stereochemistry of the product, and the outcome of deuteration experiments (*anti* stereospecificity has been considered also where it was not verified). Examples that do not fit well the model are indicated with an asterisk.

**Entry**	**EWG**	**α**	**β_1_**	**β_2_**	**Product**	**Refs**.
EWG at prochiral C, classical binding mode (Figure 3a)						
1	COCH_2_β_1_	R (Me, Et)	CH_2_CH_2_	H	(*R*)	[12]
2	COCH_2_β_1_	Me	CHRCH_2_	H	(*R*)	[13]
3	COCH_2_β_1_	OMe	CH_2_CH_2_	H	(*R*)	[14]
4	COR (Me, Et)	Me	COOR	H	(*R*)	[15, 16, 17]
5	COOR (Et, >)	Me	COOR (Et, >)	H	(*R*)	[9]
6	COOR (Et, >)	Me	CN	H	(*R*)	[18]


EWG at prochiral C, flipped binding mode (Figure 3b)						
7	CHO	R (Me, Et), OR	H	Ph	(*S*)	[19]
8	CHO	Me, Ph, NHAc	H	Ar	(*S*)	[20, 21]
9	CHO	Me	H	C≡CR	(*S*)	[22]
10	CHO	Bn	H	H	(*R*)	[23]
11	COR	R (Me, >)	H	Ar	(*S*)	[24]
12	COR	Br	H	CH_2_OR	(*S*)	[25]
13	COMe	Me	H	COOR	(*S*)	[15, 16]
14	COMe	CH_2_COOR	H	H	(*R*)	[15]
15	CO(CH_2_)_n_NHR	Me	H	Me	(*S*)	[26]
16	COOMe	CH_2_OH	H	H	(*R*)	[27]
17	COOMe	CH_2_OR	H	H	(*R*)	[28]
18	COOMe	Cl, Br	H	R, Ar	(*S*)	[29, 30, 31]
19	COOMe	R (Et, >)	H	COOMe	(*S*)	[9]
20	COOMe	R (Et, >)	H	CN	(*S*)	[18]
21	COOMe	NHCOPh	H	COOMe	(*S*)	[32]
22	CN	Ar	H	COOMe	(*R*)	[33]
23	CN	R (Me, >)	H	COOR (Me, >)	(*S*)	[34, 35]
24	CN	CH=CMe_2_	H	COOH	(*R*)	[36]
25	CN	Ar	H	H	(*R*)	[37]
26	COCH_2_β_1_	OCH_2_Ph	CH_2_CH_2_	H	(*S*)	[14]


EWG at non‐prochiral C, classical binding mode (Figure 3c)						
27	CHO	H	Me	Ph	(*S*)	[11]
28	CHO	H	Me	CH_2_CH_2_R	(*R*)	[38]
29	CHO	H	Me	C≡CR	(*S*)	[22]
30*	COMe	H	COOEt	Me	(*R*)	[39]
31	COCH_2_β_1_	H	CH_2_CH_2_	R (Me, >)	(*S*)	[12]
32	COCH_2_β_1_	H	CH_2_CH_2_	COOR (Me, Et)	(*S*)	[40,41]
33	NO_2_	H	Me	Ar	(*R*)	[42]
34	NO_2_	H	R (Me, >)	Ar	(*R*)	[43]
35	NO_2_	H	NHAc	R, Ar	(*R*)	[44]
36*	NO_2_	H	COOEt	R (Et, >)	(*R*)	[45]


EWG at non‐prochiral C, flipped binding mode (Figure 3d)						
37	CHO	H	Ph	Me	(*S*)	[11]
38*	COMe	H	Me	COOEt	(*R*)	[39]
39	COOMe	H	*i*Bu	CN	(*R*)	[34,35]
40*	COOMe, CN	H	Me	PO(OMe)_2_	(*R*)	[46]
41	COOMe	H	NHCOCH_2_Ph	COOMe	(*R*)	[32]

**Figure 3:**
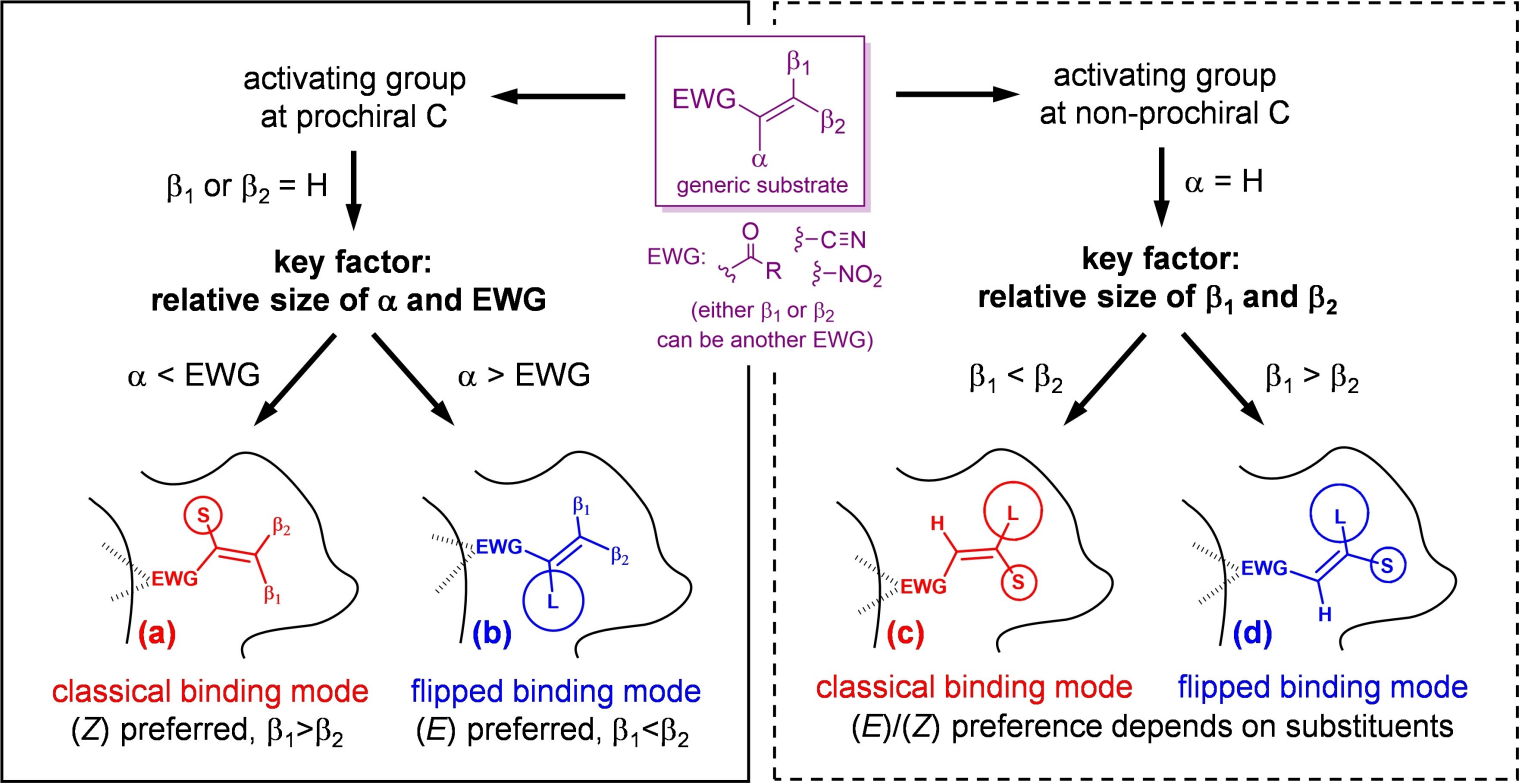
General model for the analysis of C=C bioreductions mediated by canonical OYEs (in all cases H^−^ is delivered from below the plane to the β‐position and H^+^ from above the plane to the α‐position).

Even though no predictive rule will ever replace the sound evidence of synthesising a new substrate and testing it in the laboratory, a general model that highlights the similarities between classes of well‐accepted substrates would be a useful guide to design more. To this aim, we attempted to generate a comprehensive model to rationalise the various (and often not easily predictable) experimental outcomes of the reduction of as many substrate classes as possible. Again, in the words of the great detective: *“Having gathered these facts, Watson, I smoked several pipes over them, trying to separate those which were crucial from others which were merely incidental.”* (A. Conan Doyle, *The Crooked Man*). Looking at the selection of data collected in Table 1, the general conclusions that can be drawn on the structural features of the “ideal” substrates of OYE bioreductions are shown in Figure 3. Four patterns can be distinguished depending on the identity of the activating EWG and the relative size of the substituents, leading to the preferred binding mode and stereochemistry of the substrate.

Obviously, this model constitutes only a set of very general guidelines, not a universal rule. Being inferred from a limited (albeit large) dataset, it suffers from some limitations. Firstly, it must be noted that the β,β‐disubstituted substrates allow for a number of exceptions to the model presented (right side of Figure 3, dashed box), while the behaviour of α,β‐disubstituted substrates can be predicted with higher accuracy (left side, solid box). Secondly, substrates that do not fit the model are not necessarily unreactive, but they may be accepted with more than one preferential binding mode and possibly with low selectivity, but this could be a starting point for substrate or enzyme engineering (see next section). Lastly, the data selected is based on “canonical” OYEs (*i. e*., OYE1‐3 and very closely related homologs). As described in the following section, in the last few years a large number of “new” OYEs have been identified, characterised and screened, including enantiodivergent variants, which have considerably different binding pockets. Similarly, mutations can have a remarkable effect, even inversion of stereochemical outcome; this also will be briefly addressed in the next section.

### Recent Developments in Stereoselectivity Improvement

**Figure 4:**
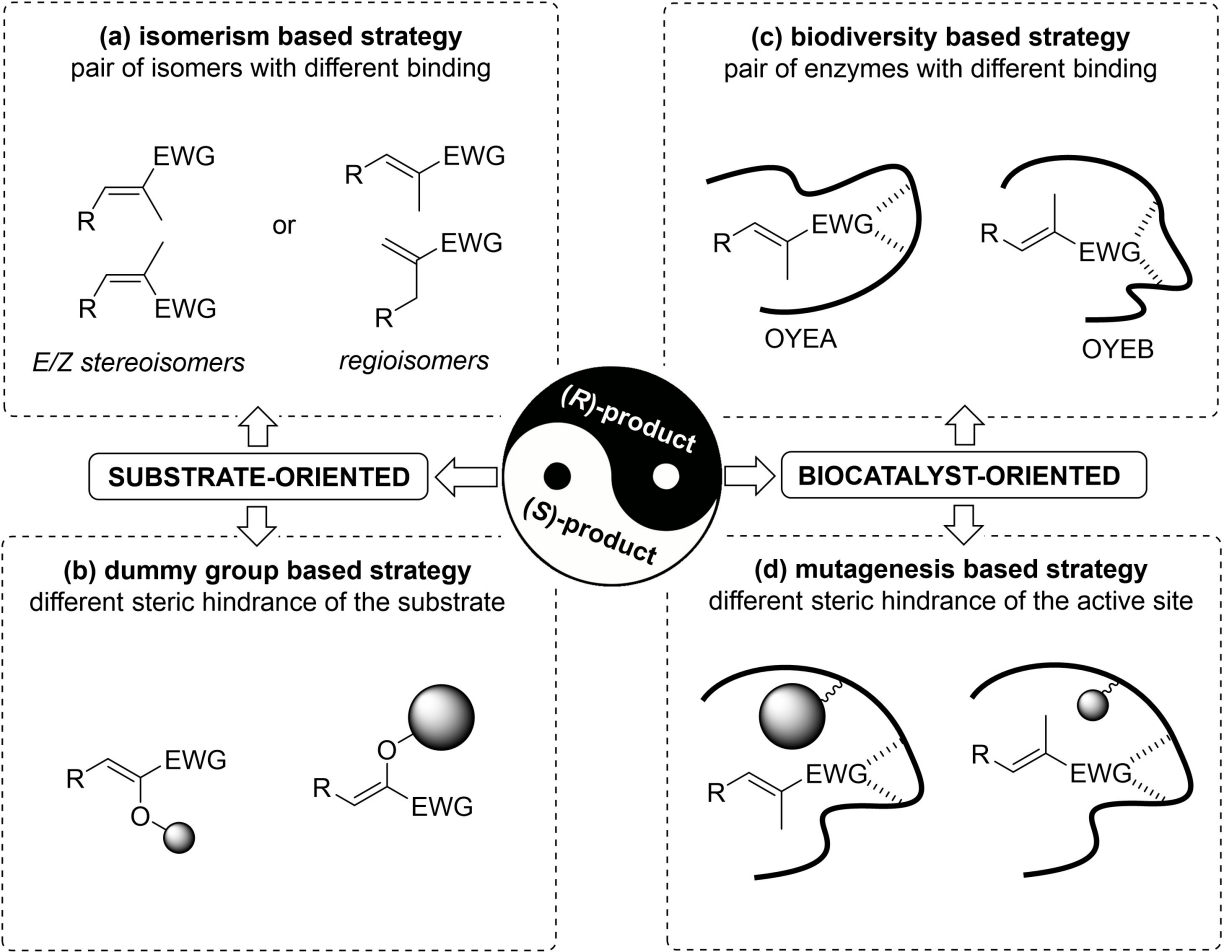
Alternative strategies for enantiodivergent reductions with OYEs (reproduced with permission from Ref. [4]).

One of the most appealing features of ER‐mediated bioreductions in biocatalytic synthesis, is their remarkable stereoselectivity, often very close to perfect, with an extremely broad range of different substrates. However, particularly in the context of pharmaceutical applications, it may happen that the optical purity of the product is insufficient and needs to be upgraded. Or it could even be the case that the bioreduction affords the opposite enantiomer of the target molecule. Fortunately, a number of strategies are available to overcome this kind of problem (Figure 4), and many successful examples have been reported in the literature of the last decade or so. Such approaches typically involve either modifications on the structure of the substrate and on the synthetic route (substrate‐oriented, Figure 4a, b) or replacing/modifying the enzyme (biocatalyst‐oriented, Figure 4c, d).

#### Isomerism‐based strategies

In some cases, the choice of a suitable pair of stereoisomeric or regioisomeric substrates (Figure 4a) can lead to perfectly opposite binding modes within the active site of the same enzyme, affording the formation of opposite enantiomers. Among the first reported examples are the diesters of isomeric citraconic, mesaconic and itaconic acids^[47]^ and the regioisomeric pair α‐methylcinnamaldehyde and 2‐benzylacrylaldehyde.^[23]^ More recently, this method has been exploited very successfully for the synthesis of both enantiomers of a family of substituted β‐ketoesters, further manipulated to yield all possible stereoisomers of a number of lactone fragrances[^15^, ^16^, ^17^] as well as in the direct reduction of regioisomeric unsaturated lactones.^[41]^

#### Dummy‐group‐based strategies

The possibility of modifying the substrate by introducing an easily replaceable “dummy” group (such as a protecting group the size of which can be decided arbitrarily by synthetic chemists) represents another exploitable strategy to influence the orientation of molecules in the active site of the enzyme (Figure 4b). In spite of the great potential of this approach, only a few examples are found in the literature.

One of the earliest reported cases was the production of both enantiomers of aspartic acid via reduction of α‐*N*‐acylamino derivatives of fumaric acid.^[32]^ The majority of acyl protective groups resulted in the formation of the (*S*)‐enantiomer with all the OYEs screened, while the largest protecting groups, such as phenylacetyl, afforded the (*R*)‐enantiomer with OYE3. Another example of this strategy is provided by the reduction of α‐alkoxycyclohexenones, where varying the size of the ether protecting group determined a switch in the stereochemical outcome of the biotransformations.^[14]^ The smallest methoxy substituent produced the (*R*)‐enantiomer, while more sterically demanding groups like benzyloxy induced the flipping of the substrate, affording the formation of (*S*)‐enantiomer, often with high *ee* values. Unfortunately, the same results were not achieved with the cyclopentenone analogues, where the formation of only (*S*)‐acyloins was achieved in all cases. Indeed, the general applicability of this strategy is limited, as a few examples are also available where it failed,^[28]^ but fortunately biocatalyst‐based approaches proved more suitable.

#### Biodiversity‐based strategies

The incredible variety of the microbiological world constitutes an almost unlimited source of new enzymatic activities. With the recent outstanding improvements in DNA sequencing technologies and bioinformatic tools, the discovery of new enzymes with novel and significantly different selectivity and activity has become remarkably easier. This applies also to OYEs, since an impressively large number of new homologs have been identified from known genomes or by metagenomic analysis of samples from disparate sources (Figure 4c). An updated table of available and well‐characterised OYE homologs has been recently published,^[8]^ although the family is expanding at an ever‐accelerating pace. The authors identified three major classes of OYEs by phylogenetic and structural analysis of known and characterised members. Class I is characterized by OYEs originating from plants and bacteria, while in class II are collected fungal OYEs that are phylogenetically and structurally closely related to the first class. Class III OYEs, instead, often present different structures, biochemical behaviours and substrate preferences and belong to a variegate group of biological sources. Thus, mining genomic and metagenomic data can afford new catalysts with enhanced or inverted stereoselectivity, and even with new activities, as illustrated with the following examples.

Extremophilic bacteria are a class of microorganisms widely investigated in order to identify new biocatalysts, and new OYE homologues with broad substrate spectrum have been isolated from radiation resistant and heavy metals resistant strains.^[48]^ The characterisation of first ER from the phylum *Basidiomycota* highlighted its ability to reduce not only α,β‐unsaturated compounds, but also activated alkynes to their saturated compounds (the enzyme being classified as an “ene/yne‐reductase”).^[49]^ In some cases, the driving force in the discovery of new ERs has been the need of new strategies for the synthesis of specific bioactive compounds: the interest for the bioreduction of chalcones lead to the identification of a new OYE homolog in the anaerobic gut bacterium *Eubacterium ramulus* ^[50]^ and, more recently, has highlighted the possibility of discovering new OYEs homologues in non‐conventional yeasts.^[51]^ Lastly, in an attempt to identify new homologs active on particularly sterically hindered substrates, an extensive data mining study on drain metagenomic samples afforded seven novel OYE‐like enzymes with activity also on bi‐ and tricyclic enones.^[52]^

The constant need for new biocatalytic tools for C=C bioreductions lead also to the exploration and characterization of non‐OYE‐like ERs, where the typical FMN is absent (nicotinamide‐dependent double bond reductases (DBRs))[^2^, ^10^, ^53^] or where it is replaced by a deazaflavin (F_420_‐dependent ene‐reductases (FDRs)).[^2^, ^54^] Due to their different binding pocket structure and geometry, such enzymes are likely to show differences in enantio‐ and regio‐selectivity, as demonstrated on specific substrate classes.[^55^, ^56^]

#### Mutagenesis‐based strategies

Courtesy of the wealth of structural and functional information available on the active sites of OYEs, the “rational design” of mutations has become routine, providing more and more variants able to satisfy the growing demand for efficient biocatalytic access to chiral molecules. Modifications of crucial amino acid residues in the active site is a powerful strategy to adjust the steric hindrance interactions and to influence the binding mode (Figure 4d). Multiple reports have been published in recent years and a comprehensive review of the structure‐guided engineering of OYEs that emerged during the last decade was published in 2020.^[57]^ Only a few representative case studies will be reported here, mainly focusing on variants with opposite enantioselectivity compared to wild‐type enzymes.

Concerning canonical OYEs, after the identification of the key role of W116 in providing stereodivergent variants in OYE1 for the reduction of carvone and related terpenes,[^58^, ^59^] the same variants were also used to afford opposite enantioselectivity in the reduction of C=C bonds in aryl‐substituted cyanoesters.^[31]^ The switch in selectivity of OYE1 W116X mutants was further explored in combination with mutants in another key amino acidic position, F296. Specifically, with α‐alkyl‐β‐arylenones, the F296S mutation combined with W116A/V provided very high stereoselectivity for the opposite enantiomer of the product with respect to wild‐type OYE1.^[60]^ Despite the very high sequence homology between OYE1 and OYE3, the same approach in mutagenesis of W116 did not lead to comparable results in inverting stereoselectivity.^[61]^

A systematic saturation mutagenesis study was carried out on OYE 2.6 from *Pichia stipitis*. Starting from 13 first‐generation, site‐saturation mutagenesis libraries, combined double and triple mutants (at Y87, I113, F247) resulted in completely reversed stereoselectivity and very high conversion on two of the three Baylis‐Hillman adducts considered in the study.^[62]^ The OYE2y homolog from *Saccharomyces cerevisiae* CICC1060 was recently engineered to obtain complete (*R*)‐enantioselectivity in the reduction of (*E/Z*)‐citral to (*R*)‐citronellal with a double mutant, albeit with a loss in enzymatic activity.^[63]^

Considering, instead, the above‐mentioned Class III OYEs, the most investigated enzyme is YqjM from *Bacillus subtilis*, which often possesses different enantioselectivity compared to canonical OYEs. For instance, a directed evolution approach afforded single and double mutants presenting high catalytic efficiency and enantioselectivity for both enantiomers of β‐substituted cyclic enones.^[64]^ Subsequently, the reduction of α‐exo‐methylene carbonyl compounds was used as a model reaction for the rational design of other YqjM mutants, leading to double variants with inverted enantioselectivity for three out of five structurally related substrates.^[65]^ Computational studies of the mechanistic steps by QM/MM methods have also been carried out to guide the future design of enantioselective YqjM mutants for other classes of substrates.^[66]^ As an alternative strategy, random or rational mutations highlighted in previous work were transferred to enzymes of the same class, to afford new mutants with enantiodivergent behaviour.^[67]^

Probably the combination of biodiversity/metagenomic based approaches (to identify the most suitable enzyme candidate), with rational design of mutations or directed evolution (to improve it up to the desired specifications) is at present the best and most reliable strategy to access the desired enantiomer of the reduced product.

## Summary and Outlook

Enzymatic stereoselective C=C reductions offer a convenient, versatile and often complementary alternative to their metal‐catalysed counterpart. The studies on the stereoselectivity of OYEs over the years enabled not only to access a broad variety of small chiral building blocks in excellent enantiopurity, but also generated a rich dataset that could be employed as the basis to formulate a general model to rationalise the preferred binding modes. Such a model may be useful to quickly sketch out the requirements of well‐established substrates and identify possible gaps. The conclusions drawn up to this point are clearly insufficient to “crack the case” of the stereoselectivity of OYEs, and to have a fully predictive model will require further extensive testing and analysis, which will be performed in the upcoming decades. As Sherlock Holmes reminds us: *“It is a capital mistake to theorise before one has data. Insensibly one begins to twist facts to suit theories, instead of theories to suit facts.”* (A. Conan Doyle, *A Scandal in Bohemia*). And this is the philosophy that should always guide all scientific endeavours.

Additionally, many different and complementary strategies have emerged to overcome the limitations of canonical OYEs, leading to higher stereoenrichment or even inverted stereopreference, by acting on substrate structure or protein sequence. These efforts will certainly contribute to enhance the efficiency and selectivity of OYE‐mediated reductions, increasing the already growing industrial interest[^68^, ^69^, ^70^] in these outstanding biocatalysts.

## Conflict of interest

The authors declare no conflict of interest.

## Biographical Information

*Fabio Parmeggiani obtained his Ph.D. in Industrial Chemistry and Chemical Engineering in Milano (Italy) in 2013, and moved to the University of Manchester (UK) to work as a research associate with Nicholas J. Turner, Sabine L. Flitsch and Jon R. Lloyd. He has been assistant professor of general and organic chemistry at Politecnico di Milano (Italy) since 2019, working in the field of biocatalysis. His current research interests include the design and exploitation of novel biocatalysts and chemo‐enzymatic cascades for industrial synthetic applications*.